# Optimal surgical population for cervical lymph node dissection in PTC

**DOI:** 10.3389/fonc.2024.1280607

**Published:** 2024-04-05

**Authors:** Yongkun Wang, Zhen Wu, Jinqiang Yan, Yumin Yao, Lin Han

**Affiliations:** ^1^ Department of Thyroid Surgery, Liaocheng People’s Hospital, Affiliated to Shandong First Medical University, Liaocheng, Shandong, China; ^2^ Department of Pathology, Liaocheng People’s Hospital, Affiliated to Shandong First Medical University, Liaocheng, Shandong, China

**Keywords:** papillary thyroid cancer, cervical lymph node dissection, metastasis, surgery, initial treatment

## Abstract

**Objective:**

There is still controversy about whether cervical lymph node dissection should be performed in surgical treatment of PTC. Based on the data of thyroid cancer patients from Liaocheng People’s Hospital from 2015 to 2018, this study focused on appropriate indications for cervical lymph node dissection surgery.

**Methods:**

The clinical and pathological data of patients with initial treatment of PTC in thyroid surgery department from 2015 to 2018 were collected. In all cases, 1001 patients underwent total thyroidectomy + central lymph node dissection, and 1107 patients underwent total thyroidectomy + central + cervical lymph node dissection.

**Results:**

The average metastasis rate of all cases was 57.23%, and even the metastasis rate of PTMC was as high as 48.97%. The total metastasis rate of central and lateral cervical lymph nodes was 74.44%, and the cervical lymph nodes were present in 49.32% of the metastatic cases. In 55.56% of the cases, the tumor diameter was more than 1 cm, and the metastasis rate of cervical lateral area was 56%. With the increase of tumor diameter, the cervical metastasis rate increased from 22.54% to 73.33%.

**Conclusion:**

The metastasis rate of PTC is more than 50%, and nearly half of them have cervical metastasis, especially in patients with high risk factors. We observed that PTC 1 cm or greater has significant rates of metastasis.

## Background

Thyroid cancer is one of the most common malignant tumors of head and neck worldwide. Thyroid cancer has a significant impact on the physical and mental health of patients and its incidence is significantly higher in women than in men (1).. Papillary thyroid carcinoma (PTC) accounts for about 80-90%, and the main route of metastasis is lymphatic metastasis (2). According to thyroid cancer diagnosis and treatment guidelines, surgical treatment is very important for the initial treatment of PTC (3). Although PTC progresses slowly, its ability to metastasize, such as to lymph nodes or even distant organs, is consistent with other malignancies (4). Generally, tumor diameter alone is not enough to determine the severity of disease (5, 6). Lymph node status is an important part of complete pathological assessment, so accurate pathological staging is more conducive to the diagnosis of patients’ disease (7). Tumor diameter is an important index of lymph node metastasis ability, and lymph node metastasis ability is also an index to evaluate tumor malignancy and invasiveness. Therefore, how to achieve standardized and accurate disease assessment of primary and metastatic lesions is of great clinical significance for postoperative treatment, rehabilitation and quality of life, and can solve the economic burden and psychological shadow of society and individuals (8).

We collected and analyzed the clinical and pathological data of 2108 patients with PTC from the Department of Thyroid Surgery and Pathology. We aim to present a relatively accurate and objective clinicopathological assessment data that may help thyroid surgeons to make a comprehensive judgment of disease staging, diagnosis and treatment.

## Methods

### Material

In this study, the clinical and pathological data of 2108 PTC patients were retrospectively analyzed, from January 1, 2015 to December 31, 2018, which underwent thyroid surgery in Liaocheng People’s Hospital. All patients were diagnosed as PTC or suspected PTC by cytopathology by fine needle aspiration before operation. All cases were eventually confirmed as papillary carcinoma of thyroid by paraffin pathology.

### Surgery

1001 patients underwent total thyroidectomy + central lymph node dissection, and 1107 patients underwent total thyroidectomy + central + cervical lymph node dissection. Informed consent of both parents for patients under 18 years old.

### Informed consent

Surgical protocols were developed in accordance with the Chinese Thyroid Association (CTA) guidelines, while respecting the experience of the surgeon. The study was conducted in accordance with the Declaration of Helsinki. The ethical approval number is 2016071.

### Data availability

Pathological data were analyzed from postoperative paraffin specimens in the pathology department. The clinicopathological information of all patients is stored in a special computer file, and a specially-assigned person is responsible for storage, input and export.

## Results

All patients were treated immediately after the lesion was found and confirmed without any observation period. Among all the cases, there were 416 males and 1692 females. The age distribution was 13-79 years old, and 285 cases were ≤35 years old (**Table 1**). 2108 cases were eventually confirmed as papillary carcinoma of thyroid by paraffin pathology, the pathological subtypes were classical (n = 1956), follicular variant (n = 65), tall cell variant (n = 28), solid variant (n = 14), diffuse sclerotic variant (n = 45).

**Table 1:** The age and gender of patients.

age	male	female	total
≤20	8	26	34
≤30	37	60	97
≤40	112	454	566
≤50	108	511	619
≤60	87	402	489
≤70	49	204	253
>71	15	35	50

The average metastasis rate of all patients who underwent either TT + CND or TT + CND + LND was 57.23%. The metastasis rate increased from 36.22% to 77.22% with increase in tumor diameter from T1a to T3 (**Table 2**). TT + CND was completed in 1001 cases, accounting for 47.48% of the total, with an average metastasis rate of 43.36%. With the increase in tumor diameter, the proportion of metastasis in central lymph nodes increased from 36.00% to 50.64%, which was not positively correlated with tumor Diameter (**Table 3**).

**Table 2:** Rate of metastasis for all patients who underwent either TT + CND or TT + CND + LND categorized by thyroid cancer diameter.

Item	Case	Ratio	Metastasis	Ratio
T1a-(≤ 0.5)	442	20.97%	160	36.20%
T1a(≤ 1)	720	34.16%	409	56.81%
T1b(≤ 2)	604	28.65%	428	70.86%
T2(≤ 4)	316	14.99%	244	77.22%
T3(> 4)	26	1.23%	17	65.38%
Collection	2108		1258	57.23%

**Table 3:** The diameter and central node metastasis.

tem	Case	Ratio	Metastasis	Ratio
T1a-(≤ 0.5)	300	29.97%	99	33.00%
T1a(≤ 1)	370	36.96%	170	45.95%
T1b(≤ 2)	235	23.48%	119	50.64%
T2(≤ 4)	85	8.49%	41	48.24%
T3(> 4)	11	1.10%	5	45.45%
Collection	1001		434	43.36%

TT + CND + LND was performed in 1107 cases, accounting for 52.52% of the total, with an total metastasis rate of 74.44% in central and lateral cervical. The central and cervical metastasis rates were 67.66% and 49.32%, respectively. With the increase of tumor diameter, the average metastasis rate (from 35.21% to 79.22%) and the cervical rate (from 22.54% to 73.33%) were gradually increased, and both were positively correlated. 42.55% of the cases had simultaneous metastasis in the central and cervical regions. In the cases with positive central lymph nodes, 62.88% had metastasis to the lateral cervical region (**Table 4**).

**Table 4:** The metastasis of cervical lymph node.

Item	Case	Central	Cervical
T1a-(≤ 0.5)	142	50	32
	12.83%	35.21%	22.54%
T1a(≤ 1)	350	221	132
	31.62%	63.14%	37.71%
T1b(≤ 2)	369	286	210
	33.33%	77.51%	56.91%
T2(≤ 4)	231	183	161
	20.87%	79.22%	69.69%
T3(> 4)	15	9	11
	1.35%	60.00%	73.33%
Collection	1107	749	546
		67.66%	49.32%

Only 25.56% of the cases had no metastasis in the cervical region and the cervical region, which was negatively correlated with the diameter of the tumor. In 25.11% of the cases, only central metastasis occurred, and the cervical lateral region was negative. Both central and lateral cervical lymph node metastasis occurred in 42.55% of cases, and the ratio increased with the increase of tumor diameter, but there was no positive correlation (**Table 5**). The rate of lymph node metastasis in T1a and below was 46.51%. The central region metastasis rate was 39.25% and the lateral neck region metastasis rate was 20.08%. The lymph node metastasis rate in T1b and above cases was 71.53%. The central region metastasis rate was 48.12% and the lateral neck region metastasis rate was 66.64%. This data was statistically significant (P<0.05). Among all the cases, 55.56% were T1b and above (T > 1cm), and the metastasis rate was 85.2%. Cases of T1b and above accounted for 63.5% of all metastatic cases, 63.82% of all central metastases, 69.96% of all cervical metastases. The proportion of cases with skip metastasis was 6.78%, that is, lymph nodes in central region were negative and lymph nodes in lateral cervical region were positive. This rate is relatively stable across all cases. Cases of T1b and above accounted for 30.67% of the total and 78.55% of metastatic cases respectively (**Table 6**).

**Table 5:** The metastasis of central and lateral lymph node (C, central; L, lateral; N, negative; P, positive).

Cervical	Cases	T1a-(≤ 0.5)	T1a(≤ 1)	T1b(≤ 2)	T2(≤ 4)	T3(> 4)
CN-LN	283	81	111	61	28	2
	25.56%	28.62%	39.22%	21.55%	9.89%	0.71%
CN-LP	75	11	18	23	20	3
	6.78%	14.67%	24.00%	30.67%	26.67%	4.00%
CP-LN	278	29	107	98	42	2
	25.11%	10.43%	38.49%	35.25%	15.11%	0.72%
CP-LP	471	21	114	186	141	8
	42.55%	4.46%	24.20%	39.49%	29.94%	1.69%

**Table 6:** The diameter and metastasis of central and lateral lymph node (C, central; L, lateral; N, negative; P, positive).

Tumor diameter (cm)	Case	CN-LN	CN-LP	CP-LN	CP-LP
T1a-(≤ 0.5)	142	81	11	29	21
		57.04%	7.75%	20.42%	14.79%
T1a(≤ 1)	350	111	18	107	114
		31.71%	5.14%	30.57%	32.57%
T1b(≤ 2)	369	61	23	98	186
		16.53%	6.23%	25.56%	50.41%
T2(≤ 4)	231	28	20	42	141
		12.12%	8.66%	18.18%	61.04%
T3(> 4)	15	2	3	2	8
		13.33%	20.00%	13.33%	53.33%

The lymph node metastasis rates of bilatera, multiple tumors and capsule invasion were respectively 65.72%,72.21% and 74.05% in all. When combined with these high-risk factors, the proportion of cervical lymph node metastasis were 55.37%,64.35% and 59.83%, respectively. According to the statistics, 193 cases of accidental contralateral malignant tumors were found, and the incidence rate was 9.16% (**Table 7**).

**Table 7:** The surgery and high-risk factors of thyroid cancer.

Item	Case	Ratio	Metastasis	Ratio	Cervical	Ratio
Bilateral	773	36.69%	508	65.72%	428	55.37%
Multiple	547	25.95%	395	72.21%	352	64.35%
Capsule invasion	483	22.91%	324	67.08%	289	59.83%
Accident Contralateral Malignancy	193	9.16%				

## Discussion

According to the SEER database of the US National Cancer Center, the incidence of PTC with diameter smaller than 1.0cm and 1.0-4.0cm gradually increased during 1980-2010, especially among the highly educated population (9). In particular, it should be noted that the possibility of dedifferentiation increases with the extension of tumor existence time (10, 11). Some studies have suggested that the degree of malignancy and dedifferentiation of PTC will increase over time (12).

Cancer is not a Chinese characteristic. Early diagnosis and treatment of cancer is the basic principle, which can significantly reduce the occurrence and recurrence risk of complications (13). Once lymph metastases occur, or distant metastases spread throughout the body, the patients will have a high risk of recurrence, and medical costs are often greatly increased (14). There is no consensus on whether prophylactic cervical lymph node dissection should be carried out (15). The academic debate focuses on the following aspects: 1. cervical lymph node dissection can increase the scope of surgical resection, enlarge the incision, and even affect the appearance. 2.cervical lymph node dissection can increase the occurrence of postoperative complications, such as shoulder syndrome, which will affect patients’ quality of life. 3.Although PTC progresses slowly, as long as it is detected in time, surgical treatment of suspicious lymph nodes can be performed without affecting the prognosis (16, 17).

The data of this study showed that the peak age of PTC was between 40 and 60 years old, and the incidence of female was significantly higher than that of male, with a ratio of 4.07:1. According to pathological data, the average lymph node metastasis rate of PTC patients was 57.23%, and the metastasis rate increased with the increase of tumor diameter, from 36.20% to 77.22%. The proportion of PTMC (diameter ≤1cm) was 47.10%, and the rate of metastasis was 46.92%, while the rate of cervical was 14.11%, which indicated that micro-carcinoma did not mean micro-invasive cancer. In 1001 cases, the metastasis rate increased from 33.00% to 50.64% with the increase of tumor diameter. In 1107 patients, the same data showed that with the increase of tumor diameter, the central metastasis rate increased from 35.21% to 79.22%, and the cervical rate increased from 22.54% to 73.33%. Among all the cases, 55.56% were T1b and above (T > 1cm), and the metastasis rate was 85.2%. Cases of T1b and above accounted for 63.5% of all metastatic cases, 63.82% of all central metastases, 69.96% of all cervical metastases. The proportion of cases with skip metastasis was 6.78%, that is, lymph nodes in central were negative and lymph nodes in cervical lateral were positive. This rate is relatively stable across all cases. Cases of T1b and above accounted for 30.67% of the total and 78.55% of metastatic cases respectively. When the tumor diameter was greater than 1 cm, the ability of metastasis the central region or even the lateral neck region was significantly enhanced, and the data of jump metastasis also increased, suggesting that the tumor diameter of more than 1 cm is an important indicator of enhanced invasiveness. When combined with the high-risk factors (bilatera, multiple tumors and capsule invasion), the proportion of cervical lymph node metastasis were 55.37%,64.35% and 59.83%, respectively. This also suggests that these risk factors were also important indicators of lymph node metastasis ability, requiring strict preoperative evaluation.

We made a statistical analysis of the data related to complications such as low calcium, hoarseness, and shoulder sensory dysfunction after PTC surgery, which will be presented in a future report. The clinicopathological features of PTC mean that the impact of treatment on survival and prognosis will be a long-term project, and we will continue to follow up these case data (18). According to CTA, all patients with PTC need long-term endocrine suppression therapy (19, 20). Radiotherapy I^131^ is also an important part of PTC comprehensive therapy (21, 22). All patients with lymph node metastases, whether central or cervical, were treated with 80-130 mCi doses of I^131^.

## Conclusions

The data from our department inform several important points.1.PTC, even PTMC, has a high rate of local metastasis, either in the central or lateral cervical region, despite being an inert tumor. Its high aggressiveness needs our attention, indicating that PTC is not low-grade malignancy as traditionally considered, and we should give appropriate treatment. 2.When there are high risk factors such as bilateral, multiple, and capsular invasion, the risk of lymph node metastasis is increased, and can more thorough surgery be considered at this time? 3.Preventive cervical lymph node dissection is not necessary, but when the diameter of the mass reaches 1 cm, the risk of central and cervical lateral lymph nodes and jump metastasis is significantly higher.

## Data availability statement

The original contributions presented in the study are included in the article/supplementary material. Further inquiries can be directed to the corresponding author.

## Ethics statement

Written informed consent was obtained from the individual(s) for the publication of any potentially identifiable images or data included in this article.

## Author contributions

YW: Conceptualization, Writing – original draft, Writing – review & editing. ZW: Data curation, Investigation, Writing – original draft. JY: Methodology, Resources, Writing – review & editing. YY: Formal analysis, Software, Supervision, Writing – original draft. LH: Project administration, Writing – original draft, Writing – review & editing.
